# Noncommunicating Fimbrial Ectopic Pregnancy Due to Intraperitoneal Transmigration of Sperm: A Case Report

**DOI:** 10.7759/cureus.80630

**Published:** 2025-03-15

**Authors:** Alexandria L Betit, Praful G Patel

**Affiliations:** 1 Obstetrics and Gynecology, Alabama College of Osteopathic Medicine, Dothan, USA; 2 Obstetrics and Gynecology, Southeast Health Medical Center, Dothan, USA

**Keywords:** complete salpingectomy, ectopic pregnancy, fimbrial ectopic pregnancy, intraperitoneal transmigration, partial salpingectomy, transmigration of sperm, tubal ectopic pregnancy

## Abstract

Ectopic pregnancies (EPs) involve the implantation of a gestational sac outside of the uterine cavity and are commonly described based on the implantation location, with most occurring within the fallopian tubes. There are a wide variety of known risk factors for EP; however, a less commonly acknowledged hypothesis includes intraperitoneal transmigration of sperm, ovum, and/or embryos. Comparatively, there are few published case reports illustrating this phenomenon, and there have been no known published case reports supporting this hypothesis involving patients without congenital uterine abnormalities. This case describes a 26-year-old G6P1132 female with a history of salpingostomy and unilateral partial salpingectomy secondary to prior EP presenting with a noncommunicating fimbrial EP likely resulting from intraperitoneal transmigration of sperm. This patient failed medical management of fimbrial EP and underwent successful surgical intervention with diagnostic laparoscopy and fimbriectomy. This case report ultimately contributes to ongoing research supporting the transmigration of gametes or embryos as a cause of EP, particularly in situations where a noncommunicating fallopian tube may still allow implantation and fertilization. Given the rising recurrence rates with each subsequent EP, implementing complete salpingectomies instead of partial salpingectomies may help lower the risk of future occurrences, even in patients who wish to preserve future fertility.

## Introduction

Ectopic pregnancies (EPs) are the leading cause of maternal mortality during the first trimester due to rupture-induced hemodynamic instability [1]. Patients often present with non-specific symptoms, including lower abdominal pain and vaginal bleeding [2]. EPs involve the implantation of a gestational sac outside of the uterine cavity and are commonly described based on the implantation location. Most EPs occur within different regions of the fallopian tubes (>90%) but can occur in other locations such as interstitially (2-4%), in cesarean scars (<1%), in the cervix (<1%), in ovaries (<3%), and in the abdomen (~1%) [2]. Within the fallopian tubes, EPs can occur in the ampulla (70%), isthmus (12%), fimbrial region (11%), and the interstitial portion (2-3%) [3]. These tubal EPs result from abnormal embryo transport in a damaged tube [4]. Some research suggests tubal EPs result from the influence of estradiol and nitrous oxide on ciliary motility and tubal smooth muscle contraction as well as from inflammation due to smoking and/or infection inducing tubal damage or dysfunction [4]. Other risk factors include age greater than 35 years, prior history of EP, diethylstilbestrol (DES) exposure, tubal damage from prior surgeries, uterine/tubal congenital abnormalities, instillation of intrauterine contraceptive devices, and usage of assisted reproductive technologies [4,5]. Less commonly, it has been hypothesized that EPs in noncommunicating tubes may result from transperitoneal migration of sperm, ovum, and/or embryos [5]. This case presentation supports this hypothesis, illustrating a 26-year-old G6P1132 female with a noncommunicating fimbrial EP likely resulting from intraperitoneal transmigration of sperm requiring surgical intervention with diagnostic laparoscopy and fimbriectomy.

## Case presentation

A 26-year-old G6P1132 African American female patient with history of obesity (BMI = 32), epilepsy not on medication, syphilis (rapid plasma reagin negative status), herpes simplex virus, and iron deficiency anemia presented to the obstetrics and gynecology (OBGYN) office with an elevated quantitative beta-human chorion gonadotropin (b-hCG) of 126.23 mIU/mL with complaints of cramping upper abdominal pain and constipation that started three days prior to presentation. The patient had a history of two prior EPs: one three and a half years prior to presentation, for which she underwent right partial salpingectomy, and one in the ampulla two years prior to the presentation, for which she underwent left salpingostomy. She denied tobacco, alcohol, and illicit drug use, and her family history was unremarkable. At the time of initial presentation, a transvaginal ultrasound (TVUS) was completed, which illustrated no intrauterine pregnancy and a right-sided corpus luteal cyst with trace free fluid in the pelvis. A complete blood count (CBC) was unremarkable, except for a hemoglobin level of 11.0 and a hematocrit level of 33.5. Given the patient’s history of EP, stable hemodynamic status with reassuring vital signs, and an unremarkable physical examination with desired continuation of pregnancy, the cause of her pain was attributed to constipation, and she was sent home with a prescription for progesterone capsules and instructions to return for increased abdominal pain, vaginal bleeding, or cramping. Five days later, the patient’s quantitative b-hCG was 901 mIU/mL.

One week after initial presentation, the patient called the office reporting 8 out of 10 in severity abdominal pain above her umbilicus without vaginal bleeding and was referred to the emergency room (ER). She was subsequently discharged from the ER with a diagnosis of constipation and was told to return if symptoms worsened or did not resolve with an adequate bowel regimen. Four days later, she returned to the office for a repeat TVUS, which revealed no intrauterine pregnancy, no ovarian cysts or masses, and no free fluid in the pelvis.

**Figure 1:**
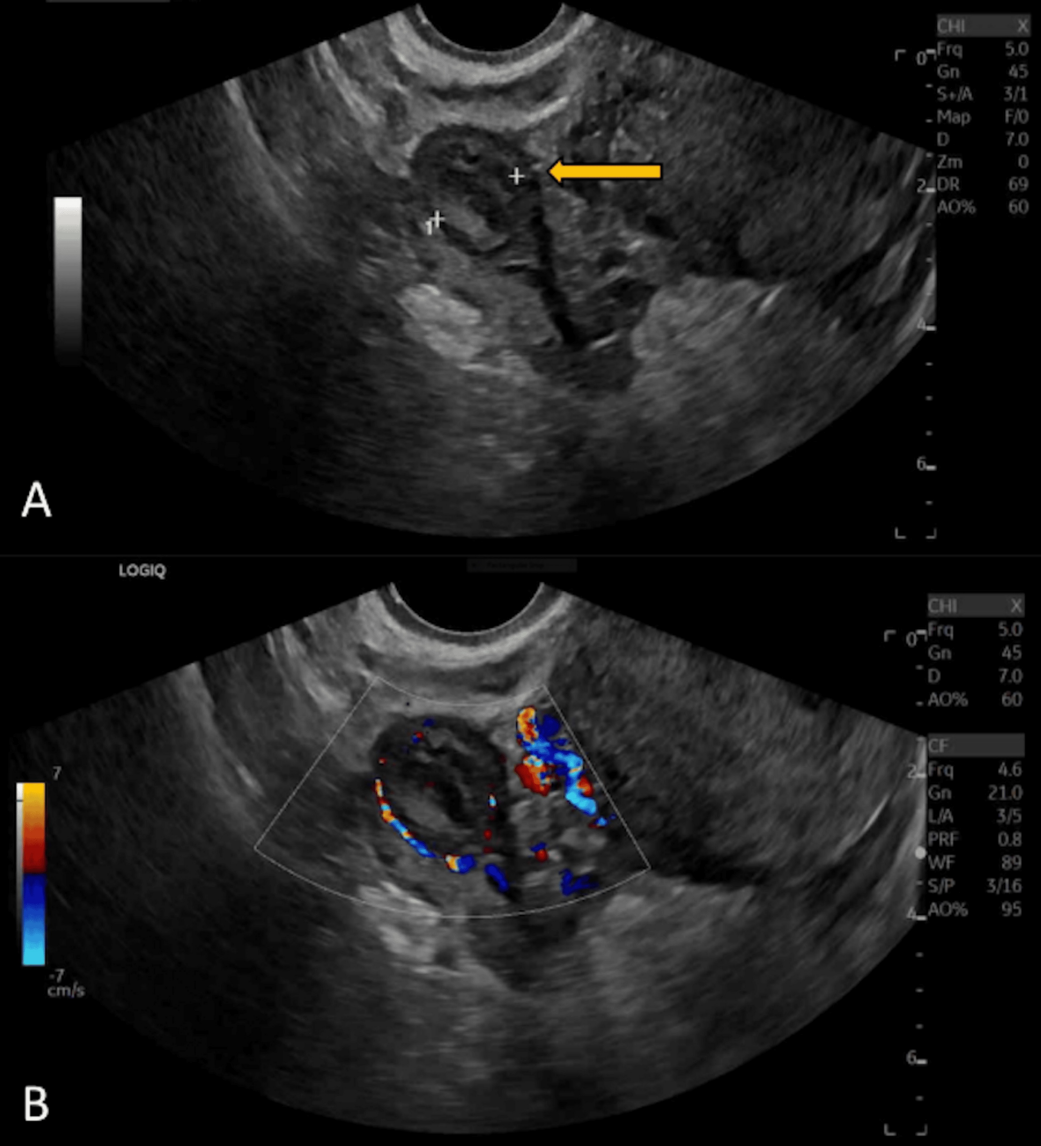
Transvaginal Doppler duplex ultrasound of the right adnexa. Figure 1A depicts a grey-scale image of a 2 x 1 cm hyperechoic area near the right ovary (yellow arrow). Figure 1B demonstrates a spectral and color Doppler image of the right ovary with a circumferential peripheral blood flow pattern characteristic of the “ring of fire” sign (white outline).

Two days later (about two weeks after initial presentation), she developed vaginal spotting and mild abdominal cramping, so she went to the ER where they performed a TVUS and labs, including a CBC and comprehensive metabolic panel (CMP), which were unremarkable, with a quantitative b-hCG of 1370 mIU/mL. The TVUS showed a 2 x 1 cm hyperechoic area near the right ovary with circumferential peripheral blood flow, which likely represented an EP (Figure 1). The patient was subsequently discharged from the ER to follow up with her OBGYN office later that same day. During her office encounter, the patient was hemodynamically stable with reassuring vital signs and had an unremarkable physical examination with no evidence of ruptured EP. Thus, medical management with methotrexate vs. surgical management with diagnostic laparoscopy for EP was discussed. The patient opted for medical management with methotrexate 90 mg intramuscular injection therapy to preserve fertility with plans to obtain quantitative b-hCG levels on days four and seven, which returned as 1855 mIU/mL and 2283 mIU/mL, respectively.

**Figure 2:**
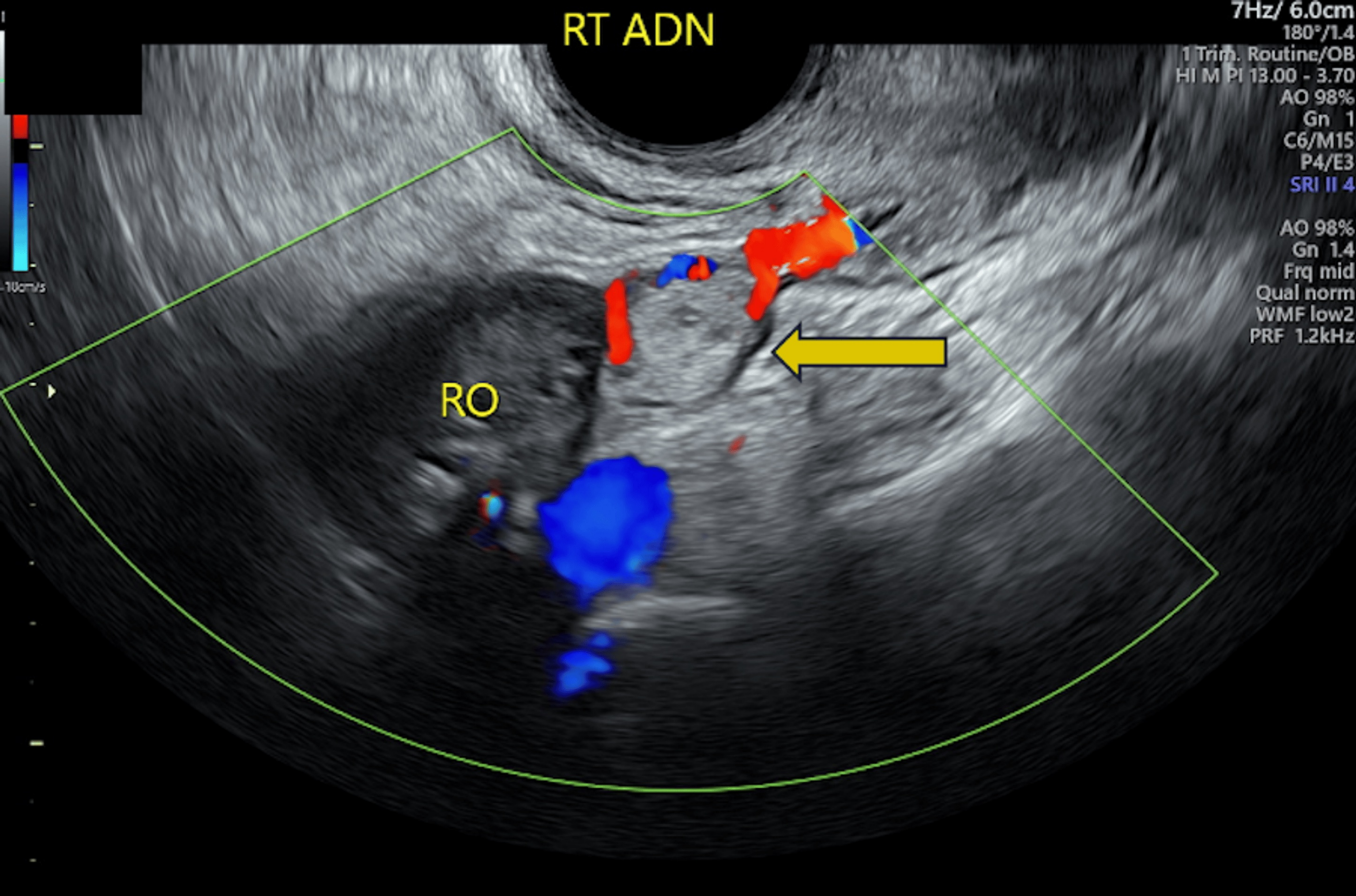
Transvaginal Doppler duplex ultrasound of the right adnexa. Complex cystic area with circumferential Doppler flow within the right adnexa measuring 1.32 x 1.20 cm without evidence of free fluid (yellow arrow). RO: right ovary; RT ADN: right adnexa.

On day seven after methotrexate therapy was administered, the patient had another TVUS, which showed no intrauterine pregnancy, benign ovaries without cysts or masses, and a 1.32 x 1.20 cm complex cystic area within the right adnexa without free fluid (Figure 2). Given the incremental rise in quantitative b-hCG despite methotrexate therapy with accompanying visualization of EP via TVUS, the patient failed medical management and elected to undergo diagnostic laparoscopy for EP treatment instead of attempting repeat methotrexate therapy. The patient was admitted to the hospital that day and underwent a diagnostic laparoscopy with fimbriectomy and chromopertubation.

**Figure 3:**
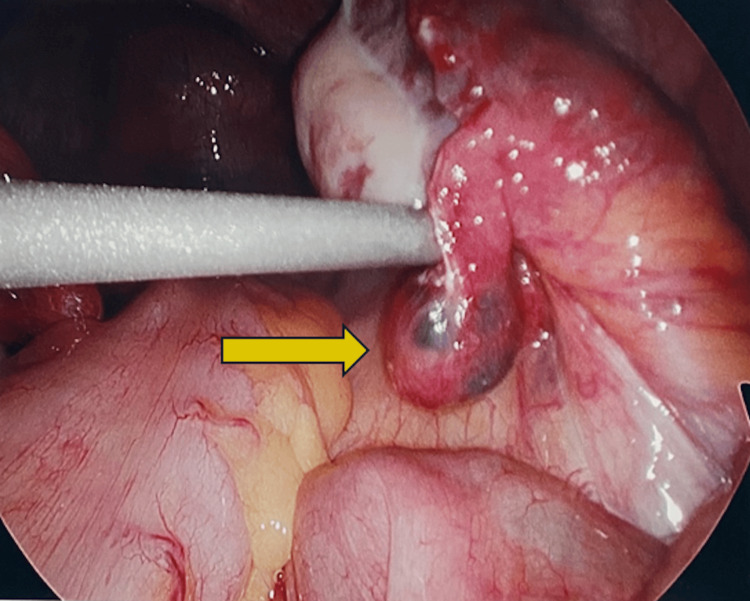
Intraoperative ectopic pregnancy in the right residual fimbria. Suction tip elevating 0.8 cm hemorrhagic nodular portion of tissue within the fimbriated portion of the right fallopian tube (yellow arrow).

Upon entering the abdominal cavity, a benign left fallopian tube and bilateral ovaries were seen, along with a small amount of hemoperitoneum. Chromopertubation revealed patency on the left and obstruction on the right. A small mass suspected to be the EP was identified on the right residual fimbria (a remnant from her prior right salpingectomy secondary to EP) (Figure 3). There was no connection noted between the right residual fimbria and the right cornu of the uterus. The EP and fimbria were removed, and the patient’s abdomen was closed. The patient was discharged home that same day, hemodynamically stable. Pathology from the suspected EP returned, indicating a grossly 0.8 cm hemorrhagic nodular portion of tissue with abundant immature chorionic villi at the fimbriated portion of a fallopian tube. The patient was seen in the office for her two-week postoperative appointment without complaints and a non-tender abdominal examination. No postoperative b-hCG levels were obtained. She remained hemodynamically stable with unremarkable vital signs throughout the course of this EP.

## Discussion

The etiology of EP has largely been equated to alterations in embryo-tubal transport and the tubal environment [6]. Yet, these mechanisms are reliant on the presence of fallopian tubes. More recently, advances in fertility treatment have increased participation in assisted reproductive technologies (ARTs). ARTs utilizing gonadotropin-releasing hormone (GnRH) agonists and those involving the transfer of fresh embryos are associated with a higher EP rate, which may be due to ovarian hyperstimulation and a hyperestrogenic environment causing poor endometrial receptivity after administration [4]. Therefore, even patients with a history of bilateral salpingectomy undergoing ART are at increased risk of EP in the cornu [7,8].

Other case reports have mentioned spontaneous EP occurrence in patients with noncommunicating fallopian tubes in conjunction with congenital abnormalities of the uterus such as unicornuate uterus and hemiuterus [5,9-13]. However, the mechanisms behind this phenomenon of EP development in patients with noncommunicating fallopian tubes have remained unproven, with hypotheses directed at the transmigration of ovum and/or sperm [5,9,10,13]. There have been no known published case reports supporting this hypothesis involving patients without congenital uterine abnormalities. In the current case presentation, the patient underwent prior left salpingostomy and right partial salpingectomy with current EP occurring in a noncommunicating right-sided fimbrial remnant. In this scenario, it is possible that a right-sided ovum was fertilized by sperm exiting from the patent left fallopian tube, which transmigrated intraperitoneally to the right residual fimbria where fertilization of the ovum occurred, resulting in the EP. This patient had no congenital abnormalities but was anatomically left with one patent fallopian tube and one fimbrial remnant. Thus, it is possible for intraperitoneal transmigration of sperm and/or ovum to occur and result in EP in cases without congenital uterine abnormalities if these patients similarly still have at least one patent fallopian tube.

It is not currently known what factors may contribute to the transport of gametes or embryos across the peritoneal cavity to reach these noncommunicating fallopian tubes [5]. However, many prior case reports suggest that a complete salpingectomy be performed rather than a partial salpingectomy in patients with prior EP when possible, given the increased risk for EP in a noncommunicating segment of the fallopian tube [5,13-15]. Considering this, the incidence of recurrent EP ranges from 6% to 28%, with the risk increasing with each subsequent occurrence [16]. Similarly, recurrence in the contralateral tube after salpingectomy is common, occurring in approximately 15% of pregnancies [17]. However, recurrence in the ipsilateral tube following partial salpingectomy is rare, with fewer than 20 cases documented before 2014 [16]. The patient described in this case report had two prior EPs and two prior surgeries but still suffered from recurrent EP at the site of a prior surgery. Given the advances in ART, reanastomosis of fallopian tubes is rarely performed [14]. Thus, in the future, we may potentially reduce the risk of EP reoccurrence by implementing complete salpingectomies in place of partial salpingectomies in cases where patients have a history of EP, even if patients desire future fertility.

## Conclusions

There are a wide variety of known risk factors for EPs; however, a less commonly acknowledged hypothesis includes intraperitoneal transmigration of sperm, ovum, and/or embryos. There have been no known published case reports supporting this hypothesis involving patients without congenital uterine abnormalities. In the current case presentation, the patient underwent prior left salpingostomy and right partial salpingectomy with current EP occurring in a noncommunicating right-sided fimbrial remnant. This case presentation supports the hypothesis of intraperitoneal transmigration of sperm as an etiologic factor in spontaneous noncommunicating fimbrial EP. The patient described in this case report had two prior EPs and two prior surgeries but still suffered from recurrent EP at the site of a prior surgery. Thus, in the future, we may potentially reduce the risk of EP reoccurrence by implementing complete salpingectomies in place of partial salpingectomies in cases where patients have a history of EP, even if patients desire future fertility.
